# Survey-based experiential learning as a new approach to strengthening non-technical skills in LMIC health care settings

**DOI:** 10.1186/s12909-021-02619-6

**Published:** 2021-04-26

**Authors:** Ruhija Hodza-Beganovic, Peter Berggren, Karin Hugelius, Samuel Edelbring

**Affiliations:** 1 School of Health Sciences, Faculty of Medicine and Health, Örebro University, Örebro, Sweden; 2 International Medical Program, Region Östergötland, Center for Disaster Medicine and Traumatology, Linköping University Hospital, Linköping, Sweden; 3 Department of Computer and Information Science, Linköping University, Linköping, Sweden

**Keywords:** Non-technical skills, NTS, Experiential learning, LMIC, Teamwork, Interprofessional learning, Design-based research

## Abstract

**Background:**

This study outlines key aspects of professional development among health professionals in low- and middle-income countries (LMIC). LMICs need support in developing their continuing medical education, and non-technical skills (NTS) that have been neglected in this respect. Given the nature of NTS, educational methods should be used experientially. This study aims to explore an interactive educational approach to increase NTS among health care professionals in an LMIC setting.

**Methods:**

A design-based research approach was applied to develop an educational method. Key NTS concepts were identified, which directed the selection of research-based surveys. A series of workshops was designed in which a survey-based experiential approach was developed. The educational process followed a pattern of individual reflection, small group discussion and relating the concepts to the local practice in a wider group.

**Results:**

An approach to increase NTS in LMIC settings emerged in iterative development through conducting workshops with health care teams in the Balkans. The topics could be grouped into individual, team, and organisational dimensions. The approach can be described as survey-based experiential learning involving steps in recurring interaction with participants. The steps include identifying concepts in individual, team and organization dimensions and contextualising them using experiential learning on the individual and group levels.

**Conclusion:**

An overarching approach has been developed that addresses NTS in an LMIC setting. The survey-based experiential learning approach can be beneficial for raising professional awareness and the development of sustainable healthcare settings in LMICs.

## Background

Low-and middle-income countries (LMICs) need support in developing their continuing medical education. This study presents an interactive educational method aiming to strengthen non-technical skills (NTS) in health care teams. Foreign Aid and donor organisations focus primarily on technical skills and equipment in LMIC. However, they are rarely adapted to the local context and thus miss the aim of long term validity for sustainable health care development This study addresses the need to develop strategies for learning NTS in continuing medical education in the context of LMICs.

To improve health care results and meet the Millennium Development Goals [1] suitable support should be provided to LMICs [2]. In order to enhance the local professionals’ capacity to contribute to a sustainable health care system, external support from aid agencies and other partners should be matched to local conditions [2]. While considerable efforts have been made to strengthen the clinical skills in LMIC, little has been done to enhance NTS [3].

Clinical skills usually focus on technical aspects and practical procedures, requiring specialised training and practice. These are necessary skills for health care professionals. However, in order for these skills to be used in an optimal way, NTS are fundamental [4]. In settings with limited resources, NTS are crucial for making full use of limited assets and collaborating effectively in teams. Consequently, scholars have emphasised the need to support health care development in LMICs in areas other than specific clinical expertise and short-sighted monetary support [5–7].

NTS are defined as cognitive and social skills, such as decision-making, planning, situation awareness, leadership, teamwork, and communication [3]. Between 50 and 80% of errors in high-risk professions can be due to professional’s behaviour related to NTS [3]. Poor team communication is frequently mentioned as a risk for surgical patient [8]. NTS educational efforts are often directed toward surgery, anaesthesia, or emergency medicine [4, 6, 9]. However, in other health care areas NTS initiatives are scarce. In many LMIC settings, training in teamwork and communication exists, but a coherent NTS training programme is generally lacking [3]. Ideally, efforts in this direction should include lifelong learning not only for physicians but also for all health professionals in an interprofessional manner. Given the characteristics of NTS, this learning should be developed through active engagement.

Despite the importance of NTS in these settings little research is available, and no context specific NTS educational tools have been found in a literature review [3]. Given the nature of NTS, such tools should be developed with a focus on experiential learning. In this study, experiential learning theory (ELT) [10] was used as a basis for an approach to sustainable educational development to strengthen lifelong learning and learning organisations. In such an approach, NTS are developed in a dialectic interplay of experience and reflection. ELT considers the experiences to consist of authentic problems, direct work life experiences and small groups of colleagues sharing and reflecting on work life experiences [11]. In response to the lack of coherent NTS training programmes, this study aims to explore an educational approach to increase NTS among health care professionals in an LMIC setting.

## Methods

This educational approach was developed using the process of design-based research (DBR), which aims to improve practice and contribute to theory [12, 13]. The educational intervention was developed through iterative cycles and refined through the joint efforts of researchers and practitioners and informed by learning theory [12]. The study was performed within a bilateral capacity building and research project supported by the Swedish Region Östergötland, tackling core principles of professional development among health professionals in the Balkans. A research team consisting of four persons with different academic backgrounds (medicine, nursing, cognitive science, and education) planned the content and the study. The SBEL development and enactment occurred in collaboration with participants in the LMIC setting. Before and after workshops, the interdisciplinary research team analysed data and interactively adjusted the steps of the next workshops, which further developed the current state of the SBEL approach.

Two two-day workshops were conducted between May 2018 and March 2019, including participants from three regions. The first workshop was conducted in Montenegro and the second in Bosnia and Herzegovina. The workshops were planned in collaboration with the participants, based on their perceived needs, to ensure that they are adapted to the local organizational cultural context.

We decided not to lecture but instead to provide an active collaborative contextualisation of the content in interaction with the participants, based on surveys as educational tools. We planned for consecutive workshops to deepen the understanding of aspects of NTS in order to support health care settings in the developmental phase in LMIC. Before and after every workshop, the research team refined and analysed the contextualised concepts in preparation for the following workshop, seeking to strike a balance between concepts and the needs and development of the participants. This iterative process formed the core of development of the approach.

### Context and participants

Twenty-nine participants with different professions from the three university hospitals in Sarajevo (Bosnia and Hercegovina), Pristina (Kosovo) and Podgorica (Montenegro) participated in the workshops. The participants were part of a long-term regional training programme [14] previously focusing on technical skills of health professionals in urodynamics and child surgery (see Table 1).

**Table 1:** Demographic data of the participants

	Workshop 1	Workshop 2
Age M (SD)	48 (8.5)	47 (8.9)
Gender (n)		
Male	7	11
Female	8	18
Profession (n)		
Physician	8	14
Nurse	7	15
Managers	3	3^b^
Country (n)		
BiH^a^	3	13
Kosovo	11	12
Montenegro	1	4

^a^Bosnia and Herzegovina

^b^Since the managers were physicians, the total number of participants is not changed

### Surveys and content of the workshops

The surveys were used as educational tools to emphasize and process NTS concepts such as situation awareness, decision-making, leadership, teamwork, and communication (see Table 2). The surveys were based on established and validated instruments. The surveys were also used to motivate and trigger reflection among participants. The surveys were translated by professional translators independently, with two translators for English–Albanian and two for English–Bosnian/Serbian. During the workshops, four translators were engaged for simultaneous translation between participants and researchers.

**Table 2:** Categorisation of professional dimensions in the workshop content

Survey	Professional dimensions addressed			Workshop		References
	Individual awareness	Teamwork	Organisation			
The Johari window model	X	X		WS1	WS2	Luft & Ingham [15]
Learning Style Questionnaire (LSQ)	X	X		X		Mumford & Honey [16]
Individual Development Plan (IDP)	X		X	X	X	Specifically designed for the study
Team Performance Observation Tool (TeamSTEPPS)	X	X	X	X		Baker et al. [17]
Team member exchange quality scale (TMX)	X	X		X		Seers [18]
Interprofessional education collaborative (IPEC)	X	X	X	X	X	IPEC [19]
Kolb’s lemon exercise, experiencing and thinking	X			X		Kolb [20]
Evaluation of the teamwork workshop	X	X	X	X	X	Specifically designed for the study

### The survey-based experiential learning approach

SBEL is an innovative educational approach using surveys to strengthen NTS in LMIC settings. SBEL consists of several steps performed in iterations in different steps, as described below.

#### Individual relation to the concepts

First, the selected concept was shortly introduced by one of the researchers. Then, the survey related to the concept was handed out to each participant, who was given time to reflect and respond on an individual basis.

#### Contextualisation in small groups

The next step was to form small groups with five to eight members pertaining to similar contexts. The purpose of this step was to share, discuss responses, and reflect on the different understandings of the concepts and how they were used in the participants’ daily work environments and organisation.

#### Contextualising in the larger setting

The third step was conducted together with all workshop participants. The small groups shared and discussed their perspectives of the concepts in relation to a broader perspective, both geographically and from point of view of the health care organisation. One of the researchers moderated the discussion to direct the participants’ perceptions toward similarities and differences.

#### Long-term development of professional awareness

The fourth step involved two parts occurring after the workshop: i) the individual’s implicit long-term conceptual understanding of and reflection upon the concepts and experiences of each participant individually or from the work groups and ii) the researchers’ continued synthesising of the contextualised understanding, which was then presented during the next workshop.

These four steps describe an iteration that was repeated for several concepts. Throughout the workshops interaction between participants and researchers supported a shared understanding.

Ethical approval was granted by the ethical committee Pristina University (ref nr. 4963).

### Analysis

Data consisted of observation of discussions, team interaction, group work, field notes, post workshop conclusion, and reflection among the research team. We did not use survey outcomes per se in this study, hence no analysis was performed on survey data. The workshop content and activities were analysed and categorized into three dimensions used to provide further structure.

## Results

The workshops were work-intensive, focusing on the participants’ active reflection on topics related to their contexts and sharing of experiences. Three dimensions emerged in the analysis of content and activities: the individual, team, and organisation (ITO). An overarching mapping of the different surveys in relation to ITO dimensions is presented in (see Table 2).

The individual dimension was addressed by all the surveys and tools. When using tools such as the Johari window and Learning style questionnaire (LSQ), each participant shared his or her understanding of the concepts on a personal level. Individuals reflected on the possible use and wording of LSQ and Johari’s exercise, and it was evident that the nurses were eager to reflect on and interpret their perceptions of the concepts. The participants became aware of how their personal strengths and limitations related to teamwork and patient care.

The individual’s role and responsibility were addressed through three surveys: individual development plan, interprofessional education collaborative (IPEC) domain descriptions and the TMX scale. These surveys focused on the individual’s role, one’s role in a team, perspectives on working as a team and working in a multi-professional team. In general, individuals shared the same view that working as a team is much easier and safer for both the patient and the professionals. The concepts of role and responsibility led to exchanges of ideas, which, according to the participants, clarified individual roles and responsibilities.

The team dimension was observed both in topics and activities. An important setting for developing the participants’ awareness of the concepts was the small group with familiar colleagues. Once the participants’ individual professional awareness on the topics was addressed, they shared experiences and understanding with colleagues in the small group setting. This interaction led to reflections in the small group on how the subject was perceived. Communication, teamwork, and reflection were important skills for professional and competent clinical practice. Sharing opinions with participants with the same profession was favoured, even if they belonged to different organisations. Both nurses and doctors were eager to discuss the concepts with colleagues of the same profession. Senior colleagues led the discussions.

TeamSTEPPS, TMX scale and IPEC addressed the team level of professional development. Concepts were related to changing health care needs and optimisation of the knowledge available in the organisation. According to the participants, traditional individual-oriented teaching should be extended by problem-based learning. Statements from IPEC domains were related to the professionals’ roles and responsibilities and sharing of responsibilities related to patient treatment. The team should be considered a safe zone since mutual agreement on the patient’s treatment is reached by the group, and the responsibility should be shared by the team. Discussion on the readiness to take over colleagues’ responsibilities in the case of emergency and to organise the work according to the available resources included several ideas, such as regular meetings to discuss tasks and engage patients and families in the treatment.

The concept of communication was evident in most of the surveys. Here, both content and interaction patterns were addressed: nurse–nurse, nurse–physician, physician–physician and communication directly with the patient.

The organisational dimension was expressed in topics and in discussions in which the participants’ various settings were compared. In the first workshop, the participants were introduced to an organisational example from Swedish health care. It was emphasised that change processes require time and adjustments. This information allowed the research team to adjust the curriculum for the workshops. Specifically, more emphasis was given to the practical application of NTS in the participants’ local contexts. For example, the participants were invited to contrast their own experiences with the presented Swedish health care setting.

In the second workshop, three managers presented their organisational settings in more detail. The presentation focused on history, the current situation, and changes since the last workshop. In addition, a site visit was planned, but unfortunately it had to be cancelled. Following the organisation presentation, participants discussed organisation and management in the full group setting. According to some group discussions, hierarchies in the workplaces was a challenging factor when establishing teamwork practices. This included both the organisation structure as well as informal roles and gender structures. In some cases, the organisation and political management structures were not supportive of interprofessional teamwork. While the participants expressed an interest in collaborating with other clinics/institutions, they lacked organisational support in the form of formal policies.

To support the long-term contextualisation, the second workshop started with a recapitulation and discussion of NTS concepts. Both understandings of the concepts and explicit observations of the concepts in one’s own workplace were addressed. The participants had developed awareness of the concepts and provided numerous examples and experiences. Some of the participants’ ideas were transformed to examples: establishing protocols and guidelines, having regular meetings and support group discussions with the members of the multi-professional team. Another aspect of long-term contextualisation was the research groups’ increased awareness of the participants’ understandings of interprofessional teamwork and challenges in the clinics.

The presentations from the three local organisations (above) were an important part of the long-term contextualisation. To further emphasise the concepts of communication and shared understanding of the local organisations, the participants were divided into three groups and asked to present their understanding of the organisations. Variations in understandings of the same organisations contributed to increased organisational awareness.

## Discussion

Overall, the findings suggest that NTS are important for professional sustainable development. Through a deeper understanding, individual reflection and team discussions, the participants saw ways this could be seen and introduced into the local organisation. Previous efforts to introduce NTS concepts in LMICs have often been developed in high-income countries and not adjusted to the context of LMICs [3]. To the best of our knowledge, this study is the first to describe an educational approach to target the development of NTS throughout all ITO dimensions in an LMIC context.

### Using SBEL approach in a LMIC setting

The health care services in LMICs are increasingly interested in creating possibilities for continuing education so that the services that are offered are efficient and reliable [2]. Efforts in this direction should target lifelong learning, not only for physicians but also for all health professionals in an interprofessional manner. SBEL involves an explicit educational approach to using surveys. Established instruments were used, not primarily for gathering data but rather for initiating reflection and discussion, inducing a shared experience in relation to the participants’ contexts. The SBEL educational approach was found to have the advantages of presenting solutions or concepts as well as stimulating continuing professional development on several organisational levels. It was modelled with consideration of the participants’ existing knowledge and practices, supporting the structure of the health care services. The necessity of shaping the interventions to the local context has been addressed in global education research [6], and the SBEL emphasises integrating aspects identified in the NTS research literature with the context, thus making this intervention innovative in terms of providing sustainable and ecological validity of the concepts in LMICs.

We sought to ensure that a broad range of relevant professions were represented in the workshops, as this was found to be important in other contexts when teaching professional awareness and skills. This was in line with DBR, where collaborative and multidisciplinary interventions between practitioners and researchers are developed in iterative and cyclic processes [13].

### Individual, team, and organisation dimensions

The identified way of structuring the range of NTS concepts into ITO dimensions proved to be supportive for organising the content into a meaningful workshop discussion. We did not seek to adjust NTS to any of the specific ITO dimensions but rather to motivate importance of understanding the dynamics of multi-professional development in an organization. The individuals in this study processed concepts by using surveys in an experiential learning manner. The Johari exercise challenged the participants into an increased individual awareness both on personal and professional levels. This awareness provided a foundation and catalysed following activities. Differences in individuals’ understandings of the concepts brought different perspectives to the multi-professional team discussions.

In the small group setting, the interactive reflections on the content of the surveys and topics were supported by the research team to facilitate the understanding of how they could be applied to the local context. According to Kolb’s learning theory, small group reflection is important for trustful communication in a group [21]. The discussion with colleagues or professionals was facilitated by the participants’ trust in each other and openness to reflect and share their experiences and ideas. Teamwork and communication skills were evident in almost all processing of surveys.

A lack of financing and human resources in LMIC health care might hinder the implementation of NTS practices [6]. This study suggests that these obstacles can be mitigated through relatively inexpensive NTS practices and training, integrating different stakeholders and supporting sustainable development.

In the larger setting, the participants applied their achieved awareness to the organisational level. Individuals’ own interpretations of NTS were distributed among the three ITO dimensions. A shared view on NTS can support a collective organizational awareness, and this can be reached through experiential learning activities [10]. The managers learned from reflecting about their organisations. They were encouraged to present information about their own organisation and to contrast it with information about the other’s organisations. Flin and O’Connor [22] argue that the presence of managers (representing leadership) as participants in training efforts deepens the discussion and dialogue by providing experience from the managerial level. Decision-making is mentioned in the NTS literature such as in the review by Scott et al. [3]. This concept was not a leading concept in our approach but was addressed in several of them such as TMX and TeamSTEPPS. The process of decision-making requires collaborative engagement and reflection which provides a critical review of its consequences [20].

### Long term development of professional awareness

The participants’ knowledge on NTS developed between and after the workshops, based on their work practice. Consequently, the SBEL approach encouraged the individuals’ implicit conceptual understandings of and reflection upon NTS in relation to their experiences. According to Kolb & Kolb [20] the long-term development of professional knowledge should be embraced in relation to the workplaces’ dynamics, social and cultural contexts, which further transform these concepts.

### The SBEL approach as an educational method

The overall goal of the SBEL approach was to increase the awareness of NTS as an important part of professional development. It was considered an appropriate educational intervention providing holistic health care education to improve patient safety.

In this study we developed an interactive educational framework for NTS to support professional awareness in an LMIC setting. The SBEL approach was inspired by Kolb’s learning theory, where the learning cycle begins with any particular action and experience, which is analysed, followed by reflection and feedback, as part of the learning experience. Kolb’s theory provides a framework for planning, implementing, and managing education actions [21]. The approach is in line with how individuals’ reason and develop their understandings in relation to their professional experiences [10]. Kolb’s theory emphasises the importance of using experiences in the learning process. The SBEL is an interactive and collaborative method, and thus, takes point of departure in the participants previous experiences. This is in line with Flin and Maran’s research on NTS [4] who consider that the understanding of NTS concepts is weaker when taught in a group without previous clinical experience. Some recommendations for further use of the SBEL approach can be formulated. All SBEL steps include reflection at individual and group levels, and time for these reflections should be allowed during the activities as well as between workshop days and between workshops. As with any other learning initiative, SBEL requires dedication and time on the part of participants. When running an SBEL workshop, it is important to provide a safe and trustful learning environment for individuals in which they can focus on understanding the introduced concepts and express their own opinions. This is promoted here, as all participants are considered equal despite having different backgrounds, genders, experiences, and roles/positions.

When organisations are invited to participate, it is important that the full spectrum of team members is included, as diversity creates a challenging and interactive learning experience. Thus, this diversity should be retained in the SBEL activities. All experiences and participant contributions should be considered equally important and should be encouraged to be articulated. An overall awareness of the cultural context and differences is needed to ensure a successful process and implementation. A future step would be to apply and evaluate the SBEL approach in other LMICs in order to evaluate its general application in other contexts.

## Conclusion

The SBEL approach can be used to support professional development and NTS in LMIC settings through research-based surveys. This approach can be applied to stimulate experimental learning in order to develop NTS and increase professional awareness of the individual, team and organisational levels in LMIC settings. It could also be used for teaching other concepts and skills of continuous medical education. The approach can encourage the development of a robust and safe health care organisation. However, it is important that considerations at the individual–team–organisation levels are taken into account among participants so that selected concepts can be tailored for the local needs.

## Data Availability

Data sharing is not applicable to this article as no datasets were generated or analysed during the current study.

## Abbreviations

DBR: Design-based research
IPEC: Interprofessional education collaboration
ITO: Individual, team, and organization
LMIC: Low- and middle-income countries
LSQ: Learning style questionnaire
NTS: Non-technical skills
SBEL: Survey based experiential learning
TMX: Team member exchange
